# Association and prognostic significance of BRCA1/2-mutation status with neoantigen load, number of tumor-infiltrating lymphocytes and expression of PD-1/PD-L1 in high grade serous ovarian cancer

**DOI:** 10.18632/oncotarget.7277

**Published:** 2016-02-09

**Authors:** Kyle C. Strickland, Brooke E. Howitt, Sachet A. Shukla, Scott Rodig, Lauren L. Ritterhouse, Joyce F. Liu, Judy E. Garber, Dipanjan Chowdhury, Catherine J. Wu, Alan D. D'Andrea, Ursula A. Matulonis, Panagiotis A. Konstantinopoulos

**Affiliations:** ^1^ Department of Pathology, Brigham and Women's Hospital, Harvard Medical School, Boston, MA, USA; ^2^ The Broad Institute of Harvard and MIT, Cambridge, MA, USA; ^3^ Medical Gynecologic Oncology Program, Dana Farber Cancer Institute, Harvard Medical School, Boston, MA, USA; ^4^ Department of Medical Oncology, Dana Farber Cancer Institute, Harvard Medical School, Boston, MA, USA; ^5^ Division of Genomic Stability and DNA Repair, Dana Farber Cancer Institute, Harvard Medical School, Boston, MA, USA

**Keywords:** high grade serous ovarian cancer, BRCA1 and BRCA2 mutations, homologous recombination DNA repair, immunogenicity, PD-1 and PD-L1

## Abstract

Immune checkpoint inhibitors (e.g., anti-PD-1 and anti-PD-L1 antibodies) have demonstrated remarkable efficacy against hypermutated cancers such as melanomas and lung carcinomas. One explanation for this effect is that hypermutated lesions harbor more tumor-specific neoantigens that stimulate recruitment of an increased number of tumor-infiltrating lymphocytes (TILs), which is counterbalanced by overexpression of immune checkpoints such as PD-1 or PD-L1. Given that BRCA1/2-mutated high grade serous ovarian cancers (HGSOCs) exhibit a higher mutational load and a unique mutational signature with an elevated number of larger indels up to 50 bp, we hypothesized that they may also harbor more tumor-specific neoantigens, and, therefore, exhibit increased TILs and PD-1/PD-L1 expression. Here, we report significantly higher predicted neoantigens in BRCA1/2-mutated tumors compared to tumors without alterations in homologous recombination (HR) genes (HR-proficient tumors). Tumors with higher neoantigen load were associated with improved overall survival and higher expression of immune genes associated with tumor cytotoxicity such as genes of the TCR, the IFN-gamma and the TNFR pathways. Furthermore, immunohistochemistry studies demonstrated that BRCA1/2-mutated tumors exhibited significantly increased CD3+ and CD8+ TILs, as well as elevated expression of PD-1 and PD-L1 in tumor-associated immune cells compared to HR-proficient tumors. Survival analysis showed that both BRCA1/2-mutation status and number of TILs were independently associated with outcome. Of note, two distinct groups of HGSOCs, one with very poor prognosis (HR proficient with low number of TILs) and one with very good prognosis (BRCA1/2-mutated tumors with high number of TILs) were defined. These findings support a link between BRCA1/2-mutation status, immunogenicity and survival, and suggesting that BRCA1/2-mutated HGSOCs may be more sensitive to PD-1/PD-L1 inhibitors compared to HR-proficient HGSOCs.

## INTRODUCTION

Immune checkpoint inhibitors (e.g., anti–PD-1 and anti–PD-L1 antibodies) have demonstrated remarkable efficacy against hypermutated cancers such as melanomas, lung carcinomas and those with underlying mismatch repair-deficiency [1–3]. One explanation for this effect is that tumors with higher mutational loads harbor more tumor-specific neoantigens that stimulate recruitment of an increased number of tumor-infiltrating lymphocytes (TILs) which is counterbalanced by overexpression of immune checkpoint modulators, such as PD-1 or PD-L1 [4–7]. In support of this, recent analyses of TCGA data have implicated neoantigen load in driving T cell responses [8], and some have identified novel associations between specific genomic alterations such as polymerase e (POLE) mutations or microsatellite instability (MSI) and increased immune infiltrates and expression of immune checkpoints in hypermutated tumors [9, 10].

Approximately 50% of high grade serous ovarian cancers (HGSOCs) harbor genetic and epigenetic alterations in gene members of the homologous recombination (HR) DNA repair pathway, most commonly in BRCA1 and BRCA2 genes [11, 12]. BRCA1/2-mutation status is a favorable prognostic factor in this disease [11, 13, 14], which may be traditionally thought to be primarily due to the enhanced responsiveness of BRCA1/2-mutated tumors to platinum-based chemotherapy. However, it is possible that alternative intrinsic biologic properties of BRCA1/2-mutated HGSOCs (e.g., increased immunogenicity) contribute to the improved outcomes observed in these patients. In this regard, it has been shown that HR deficient HGSOCs (including those with BRCA1/2-mutations) depend on alternative, low fidelity mechanisms for double-strand break (DSB) repair, such as the Polθ/PARP1-mediated alternative end-joining (alt-EJ) pathway [15, 16]. DSB repair via alt-EJ utilizes microhomology at rearrangement junctions to rejoin DSBs and is mediated by the error-prone Polθ polymerase, which produces point mutations as well as random insertions and deletions (indels) at sites of microhomology [17]. Not surprisingly, BRCA1/2-mutated HGSOCs have been shown to possess a higher number of mutations compared to non-BRCA1/2-mutated tumors [18], with an elevated number of larger indels (up to 50 bp) with overlapping microhomology at breakpoint junctions [19]. Given their higher mutational load and unique mutational signature, we hypothesized that BRCA1/2-mutated tumors may harbor more tumor-specific neoantigens, and, therefore, increased tumor-infiltrating lymphocytes (TILs) [7] as well as demonstrate increased expression of the immune checkpoint modulators, PD-1 and PD-L1.

In this study, we formally evaluated the association of BRCA1/2-mutation status with neoantigen load, number of TILs and expression of PD-1 and PD-L1 in HGSOC. Furthermore, given that both BRCA1/2-mutation status and number of TILs are known favorable prognostic factors in this disease, we assessed whether BRCA1/2-mutated HGSOCs are independently associated with survival after adjusting for neoantigen load or number of TILs.

## RESULTS

### HR deficient HGSOCs exhibit higher neoantigen load compared to HR proficient tumors

Initially, we compared the neoantigen load between BRCA1/2-mutated HGSOCs versus all remaining tumors in the TCGA dataset. Prediction of neoantigen load was performed using sequencing data from the ovarian TCGA dataset which included whole-exome sequencing data from 316 HGSOCs [11]. 71 of 316 samples were excluded from our analysis because they were comprised of only single-end reads using the SOLiD platform and thus not amenable to accurate HLA typing. Inference of HLA type was successfully performed for the remaining 245 of HGSOCs, and prediction of neoantigen load was performed using a pipeline based on the NetMHCpan [20, 21] tool that predicts MHC class I binding peptides. We predicted neoepitopes individual to each tumor arising from tumor-specific somatic mutations that could generate peptides predicted to bind to personal HLA alleles.

**Figure 1:**
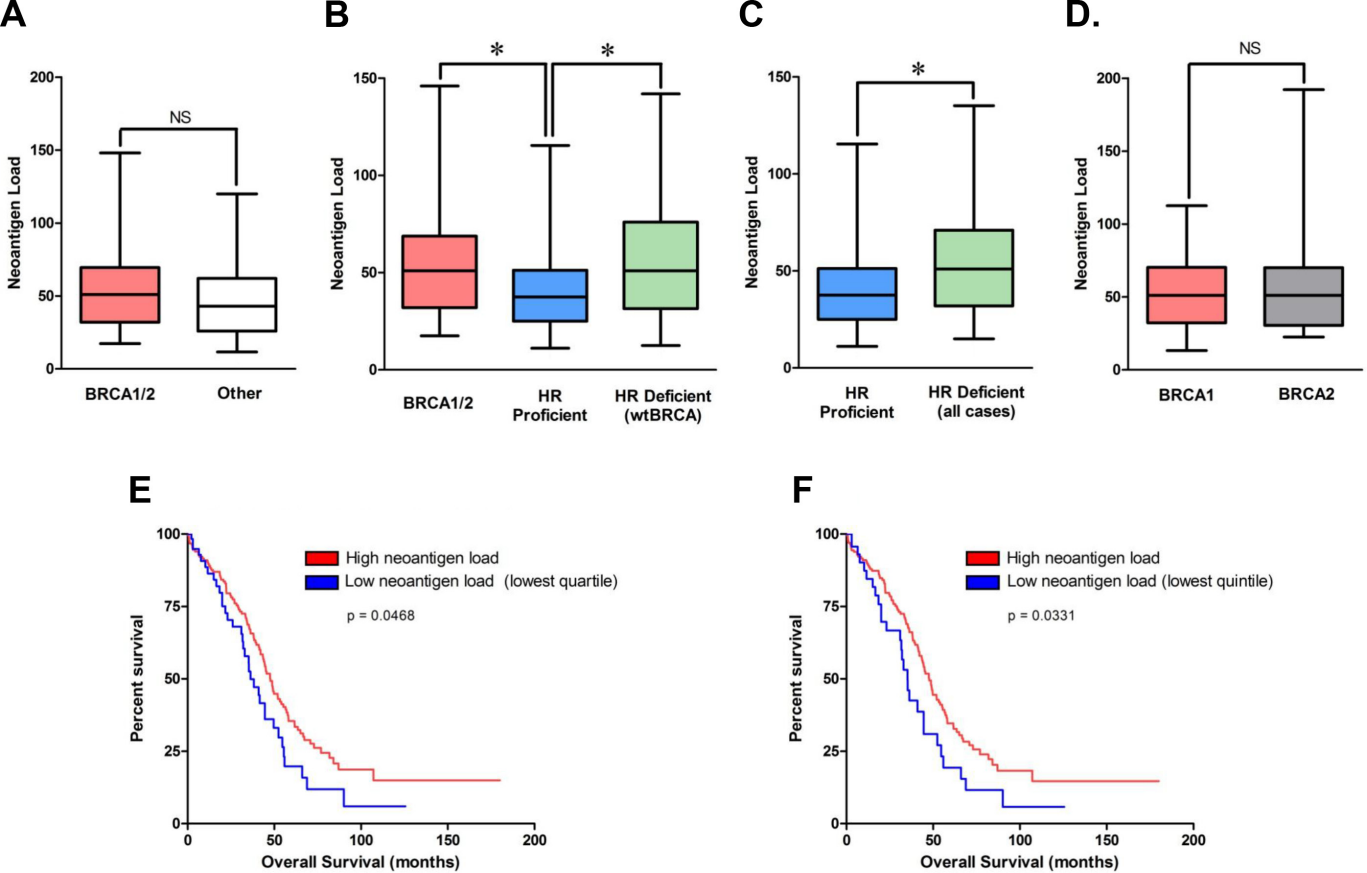
Neoantigen load in BRCA1/2-mutated, non-BRCA1/2-mutated/HR-deficient and HR proficient cohorts, and association with outcome in the TCGA dataset (**A**) Predicted neoantigen load in BRCA1/2-mutated (*n* = 54) vs all remaining non-BRCA1/2-mutated tumors (*n* = 191). (**B**) Predicted neoantigen load in BRCA1/2-mutated (*n* = 54), HR deficient/non-BRCA1/2-mutated (*n* = 69) and HR proficient tumors (*n* = 122). (**C**) Predicted neoantigen load of HR-deficient (*n* = 123) vs HR-proficient (*n* = 122). (**D**) Predicted neoantigen load of BRCA1- versus BRCA2-mutated tumors. (**E**) Tumors in the lowest quartile of neoantigen load were associated with significantly lower overall survival compared to the remaining tumors. Of the 60 tumors in the lower quartile, 20 were HR deficient and 40 were HR proficient. (**F**) Tumors in the lowest quintile of neoantigen load were associated with significantly lower overall survival compared to the remaining tumors. Of the 47 tumors in the lower quintile, 19 were HR deficient and 28 were HR proficient.

There was no statistically significant difference in the neoantigen load between BRCA1/2-mutated (germline and somatic) HGSOCs (*n* = 54) versus all remaining non-BRCA1/2-mutated tumors (*n* = 191) (*p* = 0.15, Figure 1A). However, it is now well established that some non-BRCA1/2-mutated tumors may still be HR deficient due to alterations in other HR genes. Therefore, we divided non-BRCA1/2-mutated tumors into two cohorts: 1) non-BRCA1/2 mutated HGSOCs with HR pathway alterations (HR-deficient/non-BRCA1/2-mutations cohort, *n* = 69) and 2) non-BRCA1/2-mutated HGSOCs without known alterations in the HR pathway (non-BRCA1/2-mutated and HR proficient cohort, *n* = 122). The HR-deficient/non-BRCA1/2-mutated cohort included HGSOCs with mutations in Fanconi Anemia (FA) genes, mutations in core HR RAD genes (including RAD50, RAD51 and RAD54L), mutations in DNA damage response genes involved in HR such as ATM and ATR, homozygous deletion of PTEN, amplification or mutation of EMSY, and promoter hypermethylation of BRCA1 or RAD51C.

We observed a higher neoantigen load in the BRCA1/2-mutated subset (median: 51, range: 11–199) compared to the HR-proficient subset (median: 37.5, range: 2–196) (two-sided *t*-test, *p* = 0.008, Figure 1B). Furthermore, the HR-deficient/non-BRCA1/2-mutated subset (median: 51, range: 7–279) harbored a higher neoantigen load compared to the HR-proficient subset (two-sided *t*-test, *p* = 0.003, Figure 1B). Collectively, the neoantigen load of the combined group of HR defective tumors (BRCA1/2 mutated plus HR defective /wt BRCA) (*n* = 123) was significantly higher than that of HR proficient tumors (*n* = 122), median 51 vs 37.5 respectively, *p* = 0.001 (Figure 1C). Conversely, there was no statistically significant difference in neoantigen load between BRCA1/2-mutated and HR-deficient/non-BRCA1/2-mutated subsets (two-sided *t*-test, *p* = 0.76) or between BRCA1-mutated versus BRCA2-mutated tumors (two-sided *t*-test, *p* = 0.32, Figure 1D). To summarize, HR deficient tumors, either BRCA1/2-mutated or non-BRCA1/2-mutated, demonstrated significantly higher neoantigen loads than HR proficient tumors (i.e. those without BRCA1/2-mutations and without any other HR pathway gene alterations).

### Lower neoantigen load is associated with inferior overall survival in the TCGA dataset

We evaluated the prognostic significance of neoantigen load in the TCGA dataset. Strikingly, tumors with the lowest quartile (Figure 1E) or lowest quintile (Figure 1F) of neoantigen load in the TCGA dataset were associated with significantly lower overall survival (OS) compared to the remaining tumors. There was no association of neoantigen load with disease free survival (DFS) using any cut-offs. As was previously reported [11], BRCA1/2-mutated tumors were associated with improved OS in the TCGA dataset. Importantly, in a multivariate analysis including BRCA1/2-mutation status and neoantigen load, BRCA1/2-mutation status retained its prognostic significance independently of neoantigen load (Supplementary Table 1). However, neoantigen load did not retain its prognostic significance after adjusting for BRCA1/2-mutation status in the TCGA dataset regardless of the cut-off (i.e. both using low quartile and low quintile), Supplementary Table 1.

Furthermore, we interrogated the TCGA dataset to determine whether tumors with high neoantigen load also exhibited greater expression of immune genes associated with tumor cytotoxicity. Specifically, we evaluated the expression of genes in the TCR signaling pathway (CD3G, CD3D, CD3E, LCK, LCP2, CD247, HLA-DPB1, HLA-DOB, ITK, PTPRC), the IFN-gamma pathway (STAT6, TFF3, PRKCA, TGFBR2, PIM1, PRKCH, PRKCQ, IRF4) and the TNFR pathway (TRAF1, PRF1, MAPKAPK3, TNFRSF1B, CCM2, GZMB, BIRC3, MAP3K14), and we assessed whether they were differentially expressed between tumors with high neoantigen load versus those with low antigen load (lowest quartile). Indeed, we found that several genes were statistically significantly differentially expressed between tumors with high neoantigen load versus those with low antigen load (HLA-DOB *p* = 0.05, GZMB *p* = 0.011, CD3G *p* < 0.001, CD3E *p* = 0.027, CD3D *p* < 0.001, CD247 *p* = 0.003, PRF1 *p* = 0.018, LCP2 *p* = 0.007, LCK *p* = 0.023, ITK *p* = 0.012, IRF4 *p* = 0.001, PTPRC *p* = 0.026). Strikingly, each of these genes were upregulated in the tumors with high neoantigen load compared to those with low neoantigen load. Furthermore, PD-L1 was upregulated in the tumors with high neoantigen load compared to those with low neoantigen load (*p* = 0.03).

### BRCA1/2-mutated HGSOCs harbor increased CD3+ and CD8+ TILs compared to HR proficient HGSOCs

**Figure 2:**
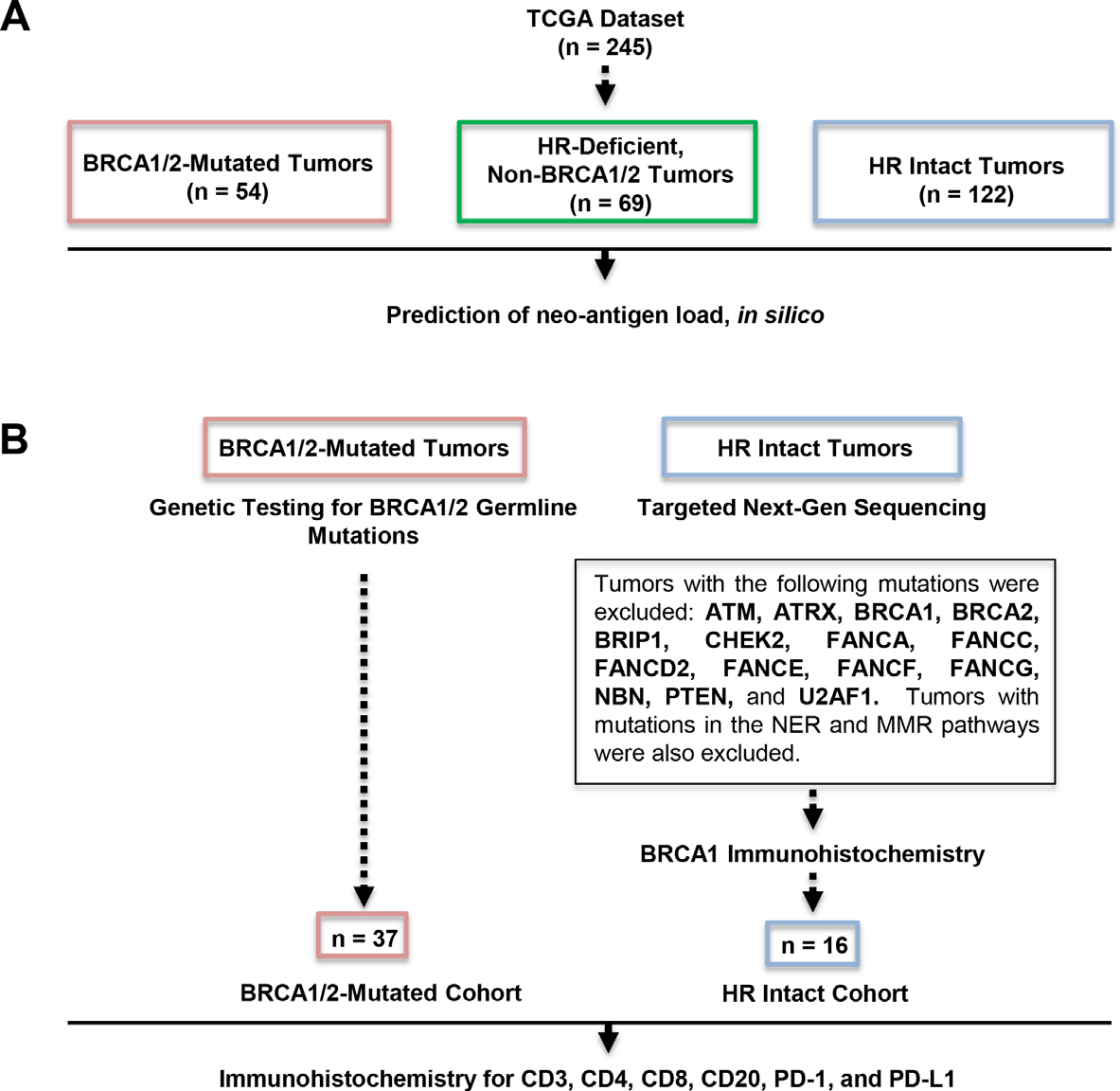
Outline of our study cohorts (**A**) Prediction of neoantigen load in the TCGA dataset. (**B**) Determination of BRCA1/2-mutated and HR proficient subsets in our institutional cohort. The BRCA1/2-mutated group was comprised of 37 HGSOCs with BRCA1/2 germline mutations (29 with BRCA1 and 8 with BRCA2 mutations) identified by genetic testing (left). The HR-proficient (HR intact) group (i.e. group without HR alterations) comprised 16 ovarian cancers which were identified in a two-step process (right). First, NGS excluded tumors with mutations in HR genes and this analysis identified 17 such tumors. Tumor was excluded based on absent BRCA1 expression by immunohistochemistry.

**Figure 3:**
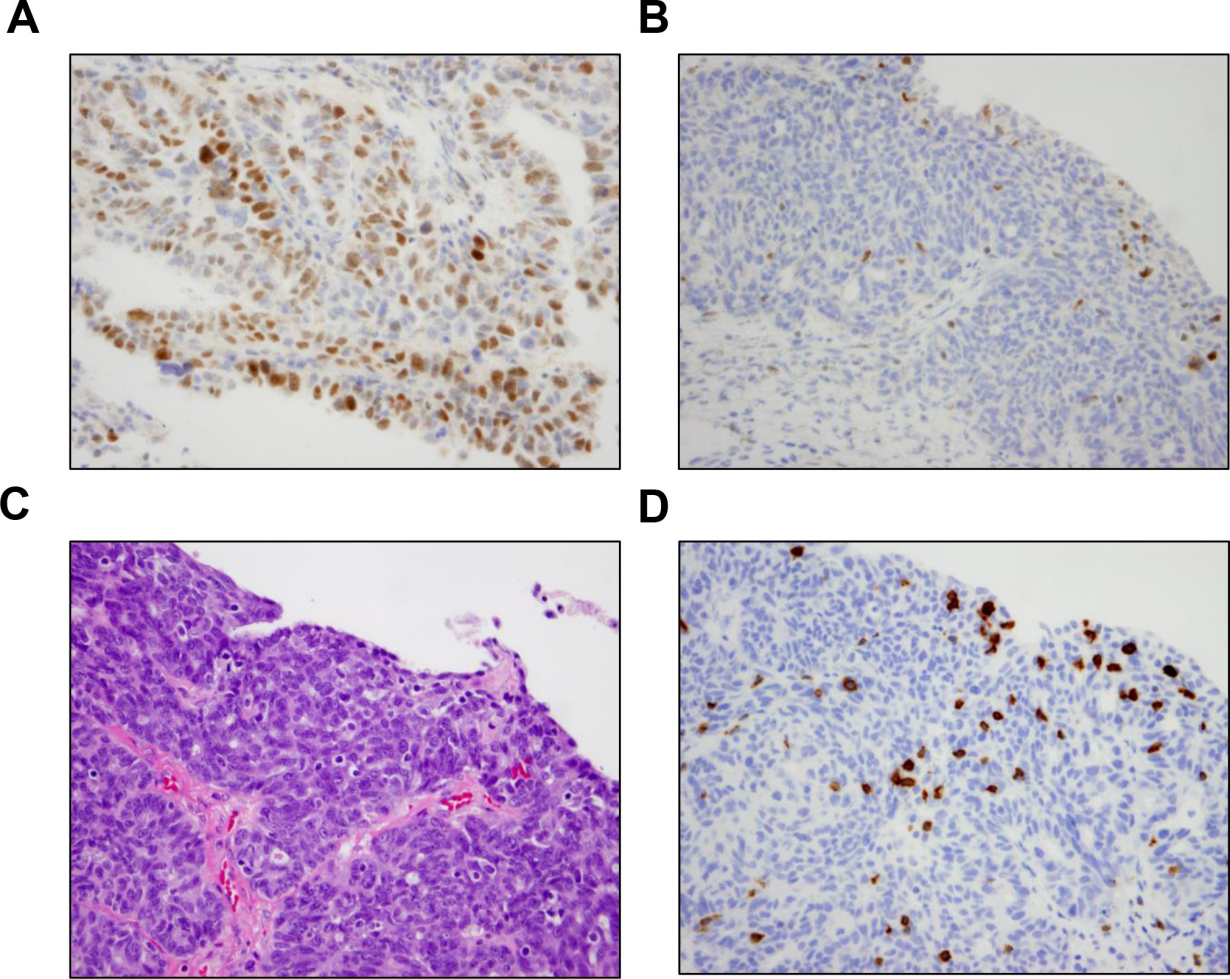
Results of BRCA1 immunohistochemistry (**A**) Positive BRCA1 IHC in a representative case. BRCA1 expression was positive by IHC in 16 of the 17 tumors without HR alterations identified by NGS. (**B**) BRCA1 IHC was negative in one tumor that did not harbor HR alterations by NGS. Focal BRCA1 positivity was present in lymphocytes. Interestingly, review of the NGS data for this case demonstrated that the tumor had a single copy deletion of the BRCA1 gene, suggesting that BRCA1 loss in this tumor was likely due to single copy deletion of BRCA1 and epigenetic silencing of the complementary allele. (**C**) Corresponding area of tumor on H & E stain demonstrates the presence of intratumoral lymphocytes. (**D**) The presence of intraepithelial lymphocytes was confirmed by a CD3 IHC.

Based on our findings from the neoantigen prediction analysis in the TCGA dataset (Figure 2A), we assessed whether there was any difference in TILs between BRCA1/2-mutated and HR proficient HGSOCs in a separate cohort of patients referred to our institution. This cohort included two groups of patients, a BRCA1/2-mutated group and an HR proficient group. The BRCA1/2-mutated group was comprised of 37 HGSOCs (29 with BRCA1 and 8 with BRCA2 mutations) with BRCA1/2 germline mutations identified by genetic testing (Figure 2B). The HR-proficient group (i.e., tumors without HR alterations) comprised 16 ovarian cancers which were identified in a two-step process (Figure 2B). First, Next Generation Sequencing (NGS) was performed to exclude tumors with mutations in HR genes, and this analysis identified 17 such tumors. These 17 tumors were subsequently evaluated for BRCA1 expression to exclude the possibility of BRCA1 promoter hypermethylation by immunohistochemistry, a method which has been previously reported to have a sensitivity of 86% and specificity of 97% for detecting loss of BRCA1 protein expression [22]. As a result of this testing, 1 tumor was found to have staining in less than 5% of tumor cells with the presence of a strong internal control (Figure 3), which was excluded from the HR-proficient (HR intact) group. Interestingly, review of the NGS data for this case demonstrated that this tumor had a single copy deletion of the BRCA1 gene, suggesting that BRCA1 loss was likely due to single copy deletion of BRCA1 and epigenetic silencing of the complementary allele. Ultimately, the HR proficient group consisted of 16 tumors without mutations in HR pathway genes and without BRCA1 loss by IHC.

**Figure 4:**
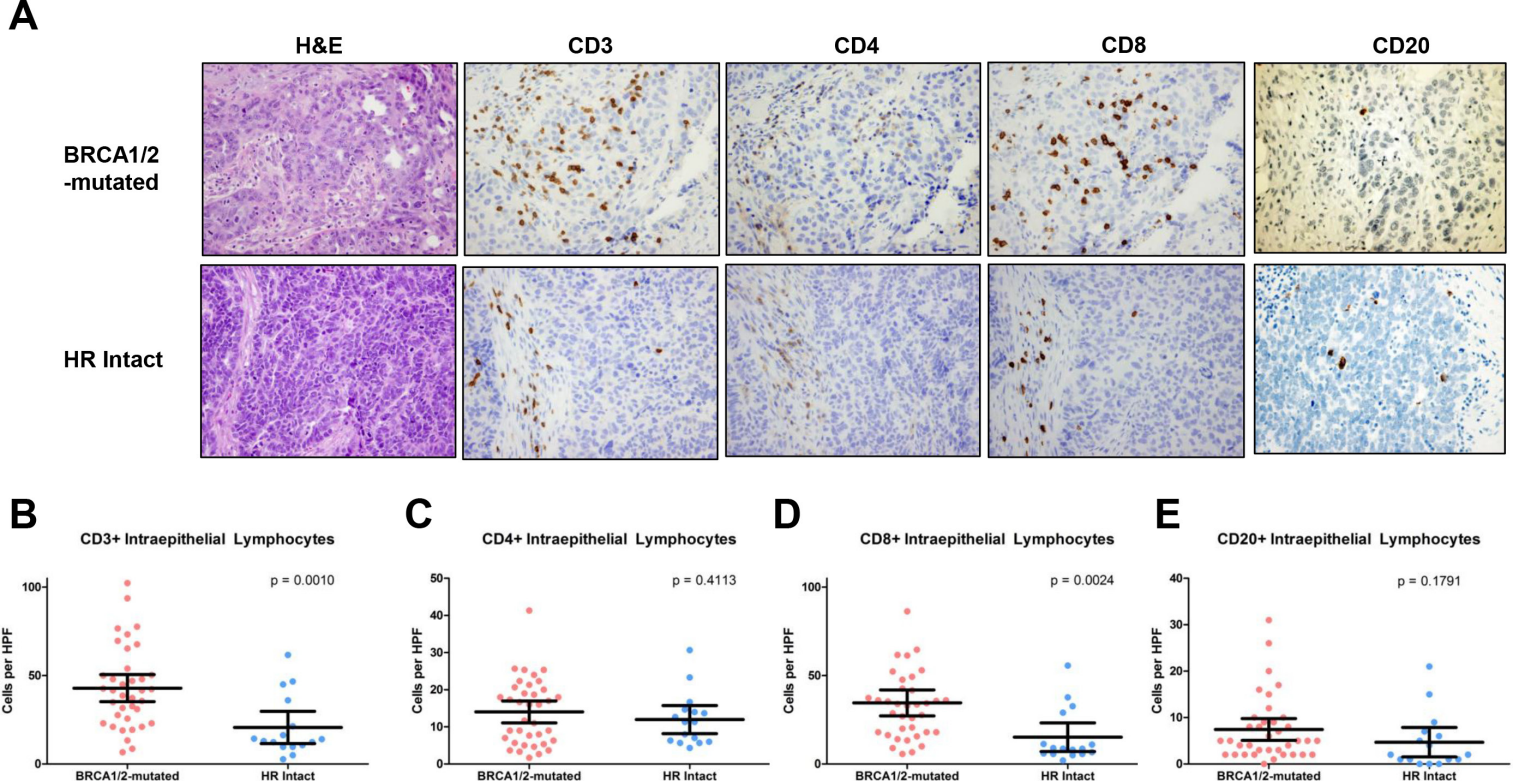
CD3+, CD4+ CD8+ and CD20+ intraepithelial lymphocytes in BRCA1/2-mutated vs HR proficient tumors (**A**) Photomicrographs of representative BRCA1/2-mutated and HR-intact tumors depicting H & E staining and immunohistochemistry for CD3, CD4, CD8 and CD20. (**B**) Quantification and comparison of CD3+ TILs from BRCA1/2-mutated and HR intact tumors. (**C**) Quantification and comparison of CD4+ TILs from BRCA1/2-mutated and HR intact tumors. (**D**) Quantification and comparison of CD8+ TILs from BRCA1/2-mutated and HR intact tumors. (**E**) Quantification and comparison of CD20+ TILs from BRCA1/2-mutated and HR intact tumors.

Immunohistochemistry (IHC) in the two patient groups demonstrated that BRCA1/2-mutated tumors exhibited a significantly higher number of CD3+ TILs (mean 42.9 vs 20.7, *p* = 0.001, Figure 4A and 4B) and CD8+ TILs (34.5 vs 15.2, *p* = 0.002, Figure 4A and 4D) compared to HR-proficient tumors. Figure 4A shows the IHC staining of a representative BRCA1/2-mutated tumor with CD3+ and CD8+ TILs, as well as a representative HR-proficient case with reduced CD3+ or CD8+ TILs. There was no statistically significant difference in CD4+ or CD20+ TILs between BRCA1/2-mutated and HR-proficient tumors (Figure 4C and 4E), but we observed a substantially higher CD8/CD4 ratio in BRCA1/2-mutated versus HR-proficient tumors (3.3 vs 1.2, *p* = 0.003). There was no statistically significant difference in CD3+ and CD8+ TILs between BRCA1 and BRCA2 mutated tumors (*p* = 0.13 and *p* = 0.63 respectively).

### BRCA1/2-mutated HGSOCs harbor increased PD-1 and PD-L1 expression compared to HR proficient HGSOCs

**Figure 5:**
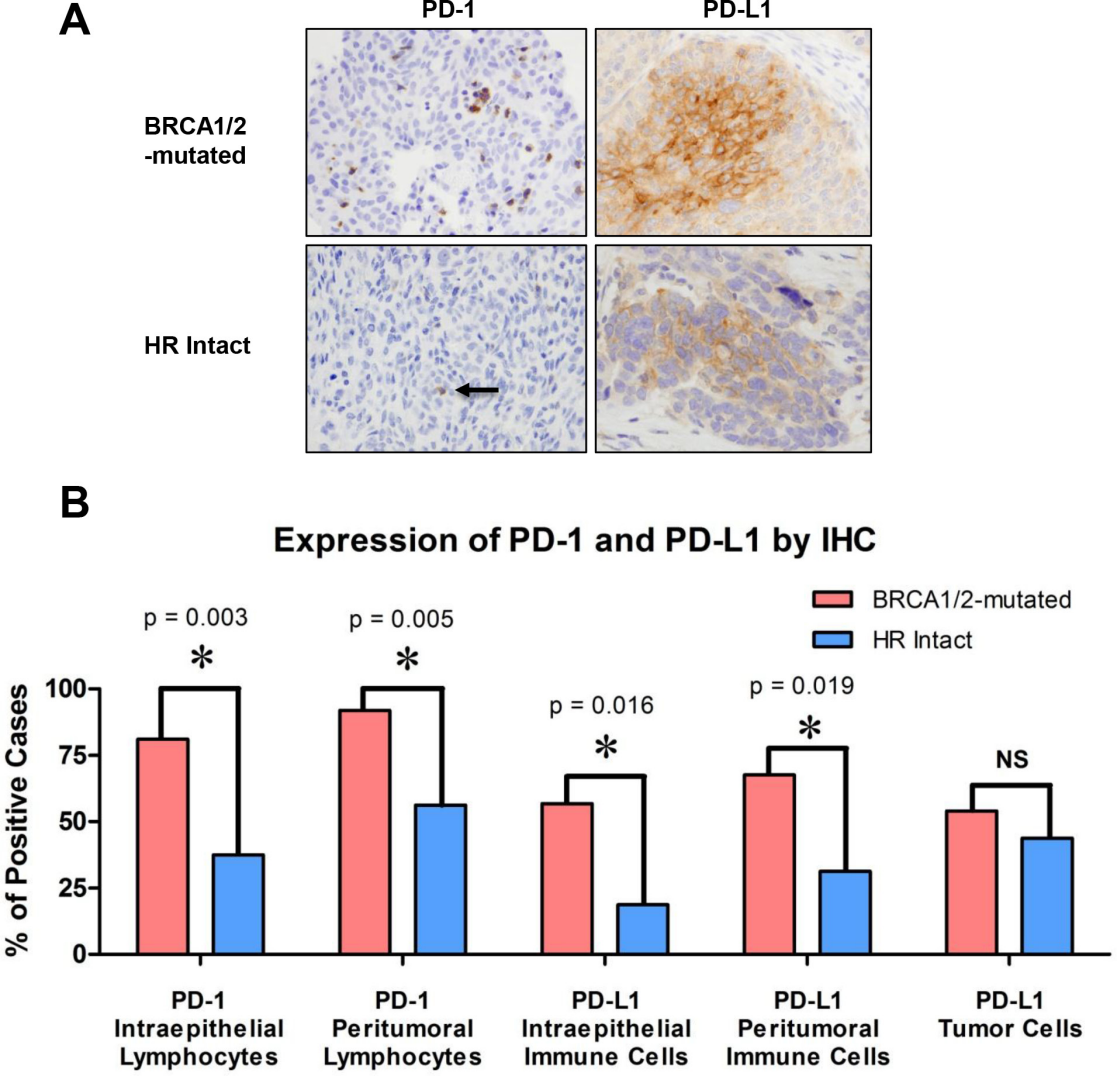
PD-1 and PD-L1 expression in the intraepithelial and peritumoral immune cells of BRCA1/2-mutated versus HR-proficient tumors (**A**) Photomicrographs of representative BRCA1/2-mutated and HR-intact tumors depicting H & E staining and immunohistochemistry for PD-1 and PD-L1. Photomicrographs depict cases from each study group that were scored as positive. (**B**) Bar graphs illustrating the number of tumors with increased PD-1 and PD-L1 positive intraepithelial and peritumoral immune cells, as well as the number of tumors positive for PD-L1 in tumor cells of BRCA1/2-mutated and HR intact cases.

We then evaluated PD-1 and PD-L1 expression both in the intraepithelial and peritumoral immune cells of BRCA1/2-mutated versus HR-proficient tumors by immunohistochemistry (Figure 5A). Expression of PD-1 in intraepithelial and peritumoral lymphocytes was significantly more frequent in BRCA1/2-mutated compared to HR-proficient HGSOCs (*p* = 0.003 and *p* = 0.005 respectively, Figure 5B). Furthermore, PD-L1 expression in intraepithelial and peritumoral immune cells was also more frequently observed in BRCA1/2-mutated tumors compared to the HR-proficient tumors (*p* = 0.016 and *p* = 0.019 respectively, Figure 5B). However, within tumor cells, PD-L1 expression was not found to be different between the two cohorts (Figure 5B). Of note, there was a significant correlation between CD3+ and both CD8+ and PD-1 positive TILs in tumors from both cohorts (Supplementary Figure 1, both *p* < 0.001).

### Prognostic significance of BRCA1/2-mutation status and number of CD3+ TILs

**Figure 6:**
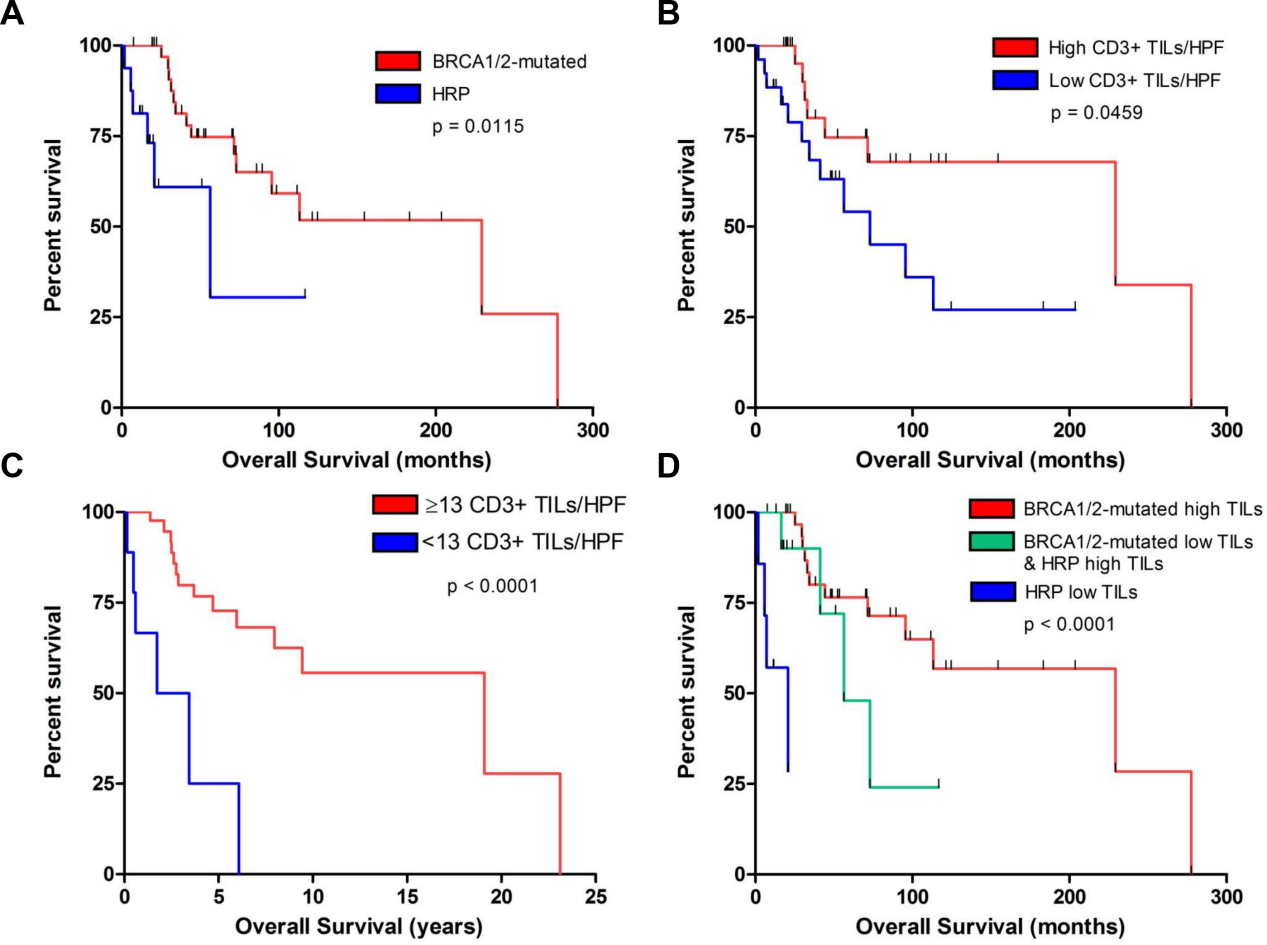
Association of CD3+ TILs and BRCA1/2-mutation status with survival in our institutional cohort (**A**) Overall survival of patients with BRCA1/2-mutated (red) versus HR intact (HRP, blue) tumors. (**B**) Overall survival of patients with tumors containing above median number of CD3+ TILs/HPF (red) versus the remaining tumors (blue). (**C**) Overall survival of patients with tumors containing ≥ 13 CD3+ TILs/HPF (red) versus those containing < 13 CD3+ TILs/HPF (blue). (**D**) Overall survival of patients with BRCA1/2-mutated tumors with a high number of TILs (≥ 13 TILs/HPF) (red), HR proficient tumors (HRP) with a low number of TILs (< 13 TILs/HPF) (blue), and BRCA1/2-mutated tumors with low TILs or HRP tumors with high TILs (green).

As expected from previous studies [11, 13, 14], BRCA1/2-mutated tumors exhibited improved OS compared to HR-proficient HGSOCs (*p* = 0.012) in our institutional cohort (Figure 6A). Furthermore, as has been previously reported, the number of CD3+ TILs was associated with survival [23]. Specifically, HGSOCs with equal or above the median number of CD3+ TILs (i.e. ≥ 35 CD3+ TILs/HPF) exhibited improved OS compared to tumors with below the median number of CD3+ TILs (i.e. < 35 CD+3 TILs/HPF) (Figure 6B, *p* = 0.046). The best discrimination for OS in our cohort was achieved using a cut-off of 13 CD3+ TILs/HPF, whereby tumors with ≥ 13 TILs/HPF exhibited significantly higher OS compared to tumors with < 13 TILs/HPF (Figure 6C, *p* < 0.001). A similar association was observed between CD3+ TILs and DFS in our cohort (Supplementary Figure 2). Importantly, in multivariate analysis consisting of BRCA1/2-mutation status and CD3+ TILs, both BRCA1/2-mutation status (HR = 0.315, 90% C.I. 0.103–0.964, *p* = 0.043) and CD3 + TILs (HR = 0.147, 90% C.I. 0.05–0.436, *p* = 0.001) remained independently associated with OS. Based on the number of TILs and BRCA1/2-mutation status, we defined a very good prognostic group (BRCA-mutated tumors and high CD3+ count, median OS 229.2 months) and a very poor prognostic group (HR-proficient tumors and low CD3+ count, median OS 20.6 months); the remaining tumors (either BRCA-mutated with low CD3+ count or with HR proficient with high CD3+ count) exhibited intermediate OS (median OS 56.3 months) (Figure 6D).

## DISCUSSION

BRCA1/2-mutated HGSOCs are HR deficient and depend on the error-prone Polθ/PARP1-mediated alt-EJ pathway for double-strand break repair [15, 16]. As a result, BRCA1/2-mutated HGSOCs possess a higher number of mutations [18, 24] with larger indels (up to 50 bp) and overlapping microhomology at breakpoint junctions [19]. Given their elevated mutational load and unique mutational signature, we hypothesized that BRCA1/2-mutated tumors may harbor more tumor-specific neoantigens, and therefore demonstrate, increased tumor-infiltrating lymphocytes (TILs) [7], as well as increased expression of immune checkpoint modulators PD-1 and PD-L1. Indeed, according to our neoantigen prediction analysis in the TCGA dataset, BRCA1/2-mutated HGSOCs exhibit significantly higher neoantigen load compared to HR proficient HGSOCs (i.e. tumors without any HR pathway alterations). Of note, HR deficient HGSOCs that were not BRCA1/2-mutated (i.e. the HR deficient/non-BRCA1/2-mutated cohort) also harbored significantly higher neoantigen load compared to HR proficient tumors. The comparatively higher neoantigen load of HR deficient HGSOCs (regardless of whether they were BRCA1/2-mutated or not) is likely related to the unique mutational signature of HR deficient tumors, which is present regardless of whether HR deficiency is due to BRCA1/2-mutations or other HR alterations [19]. In this regard, within HR deficient tumors, we observed similar neoantigen load between BRCA1/2-mutated tumors and those with alternative HR alterations (HR-deficient/non-BRCA1/2-mutated cohort), as well as a similar neoantigen load between BRCA1- and BRCA2-mutated tumors.

In addition to a low neoantigen load, HR-proficient tumors exhibited significantly lower numbers of CD3+ and CD8+ TILs, as well as lower expression of the inhibitory immune checkpoint modulators, PD-1 and PD-L1, compared to BRCA1/2-mutated tumors. This observation is consistent with the hypothesis that an elevated neoantigen load leads to an increased number of TILs that are counterbalanced by overexpression of immune checkpoint modulators [4–6]. Although PD-L1 expression in tumor-infiltrating immune cells was different between HR-proficient and BRCA1/2-mutated tumors, PD-L1 expression in cancer cells was not different. It is important to underscore that PD-L1 expression in tumor-infiltrating immune cells does not always correlate with PD-L1 expression in cancer cells. As such, response to anti-PD-L1 antibody MPDL3280A has been previously shown to correlate with tumor-infiltrating immune cell PD-L1 expression but not expression of PD-L1 in tumor cells [25]. Collectively, our findings suggest that BRCA1/2-mutated HGSOCs may be more sensitive to PD-1/PD-L1 inhibitors compared to HR-proficient HGSOCs.

Additionally, we noted that the number of CD3+ TILs significantly correlated with the number of PD-1-positive lymphocytes (Supplementary Figure 1), suggesting that, in this setting, the number of CD3+ cells could be used as a surrogate marker of PD-1 positivity. However, further investigation is required to determine if the number of TILs can predict responsiveness to anti-PD-1 or anti-PD-L1 immunotherapies. Similar to the neoantigen load prediction in the TCGA dataset, we observed no difference in the number of TILs between BRCA1- and BRCA2-mutated tumors in our patient cohort. Although previous studies have demonstrated that BRCA1 HGSOCs exhibit increased number of TILs [24, 26–28], our findings suggest that the same also applies for BRCA2-mutated HGSOCs. Most importantly, our study is the first to indicate that HGSOCs without HR alterations (HR proficient HGSOCs) represent a unique subset of tumors with lower neoantigen load, lower number of TILs and lower PD-1 and PD-L1 expression.

Finally, given that elevated TILs is a well-documented favorable prognostic factor in HGSOC [23, 29], our findings suggest that enhanced immunogenicity may also explain the improved OS of BRCA1/2-mutated tumors. Importantly, BRCA1/2-mutation status was independently associated with OS after adjusting either for neoantigen load in the TCGA dataset or for number of TILs in our patient cohort, a finding that suggests that alternative factors that are intrinsic to BRCA1/2-mutated tumors (such as enhanced response to platinum chemotherapy among other possibilities) may also contribute to the improved OS of these tumors, independently of their association with elevated number of TILs. Strikingly, BRCA1/2-mutated tumors with elevated TILs were associated with the best prognosis in our patient cohort while tumors that were both HR-proficient-tumors and had low number of TILs exhibited the worst prognosis (Figure 6D).

In contrast to the number of TILs, neoantigen load was significantly associated with OS but not PFS. It is possible that an association between neoantigen load and PFS may exist but was not observed in the TCGA dataset. Of note, only patients with neoantigen load in the lower quartile or quintile had lower OS, suggesting that additional factors are likely responsible. Furthermore, it is important to underscore that PFS is a marker of outcome that reflects more the responsiveness to first line chemotherapy and less the biological aggressiveness of the disease is (which is more globally reflected by OS). Therefore, lower neoantigen load may reflect more aggressive disease and thus inferior OS but not necessarily worse response to first line chemotherapy.

In conclusion, our findings support a link between BRCA1/2-mutation status, immunogenicity and improved survival in HGSOC, and support inclusion of BRCA1/2-mutations and other HR alterations as exploratory biomarkers in immunotherapy trials in this disease. Furthermore, our study suggests that BRCA1/2-mutated HGSOCs may be more sensitive to PD-1/PD-L1 inhibitors compared to HR-proficient HGSOCs.

## MATERIALS AND METHODS

### Prediction of HLA type and neoantigen load

Inference of HLA type was performed by applying the POLYSOLVER (POLYmorphic loci reSOLVER) tool [30] to whole-exome sequencing (WES) data generated from The Cancer Genome Atlas (TCGA) consortium as previously described [31]. Polysolver has previously been validated on a set of 253 HapMap samples with experimentally determined HLA genotypes, where it was found to have ∼97% mean overall accuracy at the protein-coding level [31]. In brief, this algorithm selects and aligns putative HLA reads to an imputed library of full-length genomic HLA allele sequences. The alignments then serve as a basis for the inference step that incorporates the number and base qualities of aligned reads, the empirical library insert size distribution and population-based allele frequencies. For prediction of neoantigen load, we used previously curated lists of somatic mutations (somatic single nucleotide variants and somatic insertions and deletions) for each of these samples (Sage Bionetworks' Synapse resource: http://www.synapse.org/#!synapse:syn1729383 and Lawrence et al. [32]) from which individual-specific HLA-binding peptides were identified by a neoantigen prediction pipeline [30] that uses detected somatic mutations in the individual. Binding affinities of all possible 9 and 10-mer mutant peptides to the corresponding POLYSOLVER-inferred HLA alleles were predicted using NetMHCpan (v2.4) [21]. All predicted binders with an affinity < 500 nM were used to evaluate the neoantigen load.

### Next generation sequencing

In order to identify tumors without HR alterations, HGSOC samples were subjected to targeted Next-Generation sequencing (NGS) assay (OncoPanel), performed at the Center for Advanced Molecular Diagnostics (Department of Pathology, Brigham and Women's Hospital) [33]. This assay has been extensively validated and is used as a CLIA-approved clinical molecular test in our institution without any additional sequencing assays to validate the findings. FFPE samples were digested in proteinase K overnight and DNA was isolated according to the manufacturer's protocol (QIAamp DNA Mini Kit, QIAGEN, Gaithersburg, MD, USA). All cases with at least 50 ng of DNA (up to 200 ng) were subjected to next-generation sequencing (NGS) of the complete exons of 275 oncogenes and tumor suppressor genes. Ninety-one intronic regions across 30 genes were also included for the evaluation of structural rearrangements. Targeted sequences were captured using a solution-phase Agilent SureSelect hybrid capture kit (Agilent Technologies, Inc, Santa Clara, CA, USA), and massively parallel sequencing was performed on an Illumina HiSeq 2500 sequencer (Illumina, Inc, San Diego, CA, USA). Mutation calls were made using Mutect and GATK software (Broad Institute, Cambridge, MA, USA) and gene-level copy number alterations at the level of individual genes were assessed using VisCap Cancer (Dana Farber Cancer Institute, Boston, MA, USA). Tumors were assessed for mutations in the following HR-pathway genes: ATM, ATRX, BRCA1, BRCA2, BRIP1, CHEK2, FANCA, FANCC, FANCD2, FANCE, FANCF, FANCG, NBN, PTEN, and U2AF1. Additionally, tumors with mutations in the nucleotide excision repair (NER) and mismatch repair (MMR) pathways were excluded from the HR-proficient cohort. We thus identified 17 tumors without mutations in one or more of these HR pathway genes.

### BRCA1 immunohistochemistry

The 17 tumors identified via NGS were subsequently evaluated for BRCA1 by immunohistochemistry to assess for BRCA1 loss due to epigenetic silencing. Immunohistochemistry for BRCA1 was performed in a manner previously described [22]. The sensitivity and specificity for BRCA1 immunohistochemistry has previously been established and found to detect BRCA1 mutations and promoter hypermethylation with 86% sensitivity and 97% specificity [22].

### Immunohistochemistry and evaluation of tumor associated lymphocytes

Paraffin-embedded, formalin-fixed tissue blocks of chemotherapy-naive biopsy and resection specimens were retrieved from the Brigham and Women's Hospital Department of Pathology archives. For all cases, IHC was performed for CD3, CD4, CD8, CD20, PD-1, and PD-L1 slides using standard protocols (Supplementary Table 2). TILs were defined as intraepithelial lymphocytes (i.e. cells that were clearly located within tumor epithelium rather than peritumoral stroma). Photomicrographs were taken of three areas enriched for intraepithelial CD3+ lymphocytes (40X objective) with blinding to mutational status. For the analysis of all markers, areas of acute inflammation and necrosis were avoided. Photomicrographs of the corresponding tumor location were obtained for CD4 and CD8 stains. Counts of intraepithelial lymphocytes were performed manually with blinding to mutation status, and the average was determined from counts of three high power fields (HPFs), as previously described [9]. A separate photomicrograph was obtained in an area enriched for CD20+ intraepithelial lymphocytes. The number of intraepithelial PD-1 positive lymphocytes was determined as the average count from three HPFs. For statistical analyses, an average of 1 or greater PD-1-positive cells per HPF was considered positive. Peritumoral T-cells were scored using a semi-quantitative system (minimal (0), mild (1+), moderate (2+), and marked (3+)), with a score of mild or greater used as a cutoff for elevated peritumoral lymphocytic response. PD-L1 in intraepithelial and peritumoral immune cells was also evaluated using a semi-quantitative scoring system (negative (0), mild (1+), moderate (2+)). Tumor cell expression of PD-L1 was evaluated in a semi-quantitatively as above, similar to methods previously described. [34] Positive tumor expression of PD-L1 was defined as greater than or equal to 5% of tumor cells with PD-L1 positivity.

### Statistical analyses

Statistical comparisons of lymphocyte counts between BRCA1/2-mutated and HR-intact tumors were performed using unpaired, two-tailed Student's *t*-test, Fisher's exact test, and Spearman correlations in GraphPad Prism (v5). Kaplan-Meier survival curves and multivariate Cox regression analyses were performed using SPSS software.

## SUPPLEMENTARY FIGURES AND TABLES



## References

[1] Brahmer JR, Tykodi SS, Chow LQ, Hwu WJ, Topalian SL, Hwu P, Drake CG, Camacho LH, Kauh J, Odunsi K, Pitot HC, Hamid O, Bhatia S (2012). Safety and activity of anti-PD-L1 antibody in patients with advanced cancer. N Engl J Med.

[2] Topalian SL, Hodi FS, Brahmer JR, Gettinger SN, Smith DC, McDermott DF, Powderly JD, Carvajal RD, Sosman JA, Atkins MB, Leming PD, Spigel DR, Antonia SJ (2012). Safety, activity, and immune correlates of anti-PD-1 antibody in cancer. N Engl J Med.

[3] Le DT, Uram JN, Wang H, Bartlett BR, Kemberling H, Eyring AD, Skora AD, Luber BS, Azad NS, Laheru D, Biedrzycki B, Donehower RC, Zaheer A (2015). PD-1 Blockade in Tumors with Mismatch-Repair Deficiency. N Engl J Med.

[4] Xiao Y, Freeman GJ (2015). The microsatellite instable subset of colorectal cancer is a particularly good candidate for checkpoint blockade immunotherapy. Cancer Discov.

[5] Llosa NJ, Cruise M, Tam A, Wicks EC, Hechenbleikner EM, Taube JM, Blosser RL, Fan H, Wang H, Luber BS, Zhang M, Papadopoulos N, Kinzler KW (2014). The vigorous immune microenvironment of microsatellite instable colon cancer is balanced by multiple counter-inhibitory checkpoints. Cancer Discov.

[6] Hussein YR, Weigelt B, Levine DA, Schoolmeester JK, Dao LN, Balzer BL, Liles G, Karlan B, Kobel M, Lee CH, Soslow RA (2015). Clinicopathological analysis of endometrial carcinomas harboring somatic POLE exonuclease domain mutations. Mod Pathol.

[7] Rooney MS, Shukla SA, Wu CJ, Getz G, Hacohen N (2015). Molecular and genetic properties of tumors associated with local immune cytolytic activity. Cell.

[8] Brown SD, Warren RL, Gibb EA, Martin SD, Spinelli JJ, Nelson BH, Holt RA (2014). Neo-antigens predicted by tumor genome meta-analysis correlate with increased patient survival. Genome Res.

[9] Howitt BE, Shukla SA, Sholl LM, Ritterhouse LL, Watkins JC, Rodig S, Stover E, Strickland KC, D'Andrea AD, Wu CJ, Matulonis UA, Konstantinopoulos PA (2015). Association of Polymerase e-Mutated and Microsatellite-Instable Endometrial Cancers With Neoantigen Load, Number of Tumor-Infiltrating Lymphocytes, and Expression of PD-1 and PD-L1. JAMA Oncol.

[10] Llosa NJ, Cruise M, Tam A, Wicks EC, Hechenbleikner EM, Taube JM, Blosser RL, Fan H, Wang H, Luber BS, Zhang M, Papadopoulos N, Kinzler KW (2015). The vigorous immune microenvironment of microsatellite instable colon cancer is balanced by multiple counter-inhibitory checkpoints. Cancer Discov.

[11] TCGA (2011). Integrated genomic analyses of ovarian carcinoma. Nature.

[12] Konstantinopoulos PA, Ceccaldi R, Shapiro GI, D'Andrea AD (2015). Homologous Recombination Deficiency: Exploiting the Fundamental Vulnerability of Ovarian Cancer. Cancer Discov.

[13] Bolton KL, Chenevix-Trench G, Goh C, Sadetzki S, Ramus SJ, Karlan BY, Lambrechts D, Despierre E, Barrowdale D, McGuffog L, Healey S, Easton DF, Sinilnikova O (2012). Association between BRCA1 and BRCA2 mutations and survival in women with invasive epithelial ovarian cancer. JAMA.

[14] Boyd J, Sonoda Y, Federici MG, Bogomolniy F, Rhei E, Maresco DL, Saigo PE, Almadrones LA, Barakat RR, Brown CL, Chi DS, Curtin JP, Poynor EA (2000). Clinicopathologic features of BRCA-linked and sporadic ovarian cancer. JAMA.

[15] Ceccaldi R, Liu JC, Amunugama R, Hajdu I, Primack B, Petalcorin MI, O'Connor KW, Konstantinopoulos PA, Elledge SJ, Boulton SJ, Yusufzai T, D'Andrea AD (2015). Homologous-recombination-deficient tumours are dependent on Poltheta-mediated repair. Nature.

[16] Mateos-Gomez PA, Gong F, Nair N, Miller KM, Lazzerini-Denchi E, Sfeir A (2015). Mammalian polymerase theta promotes alternative NHEJ and suppresses recombination. Nature.

[17] Yousefzadeh MJ, Wood RD (2013). DNA polymerase POLQ and cellular defense against DNA damage. DNA repair.

[18] Birkbak NJ, Kochupurakkal B, Izarzugaza JM, Eklund AC, Li Y, Liu J, Szallasi Z, Matulonis UA, Richardson AL, Iglehart JD, Wang ZC (2013). Tumor mutation burden forecasts outcome in ovarian cancer with BRCA1 or BRCA2 mutations. PLoS One.

[19] Alexandrov LB, Nik-Zainal S, Wedge DC, Aparicio SA, Behjati S, Biankin AV, Bignell GR, Bolli N, Borg A, Borresen-Dale AL, Boyault S, Burkhardt B, Butler AP (2013). Signatures of mutational processes in human cancer. Nature.

[20] Shukla SA, Rajasagi M, Tiao G, Dixon PM, Lawrence MS, Stevens J, Lane WJ, Dellagatta JL, Steelman S, Sougnez C, Cibulskis K, Kiezun A, Brusic V (2015). Comprehensive analysis of cancer-associated somatic mutations in class I HLA genes. Nat Biotechnol.

[21] Nielsen M, Lundegaard C, Blicher T, Lamberth K, Harndahl M, Justesen S, Roder G, Peters B, Sette A, Lund O, Buus S (2007). NetMHCpan, a method for quantitative predictions of peptide binding to any HLA-A and -B locus protein of known sequence. PLoS One.

[22] Garg K, Levine DA, Olvera N, Dao F, Bisogna M, Secord AA, Berchuck A, Cerami E, Schultz N, Soslow RA (2013). BRCA1 immunohistochemistry in a molecularly characterized cohort of ovarian high-grade serous carcinomas. Am J Surg Pathol.

[23] Zhang L, Conejo-Garcia JR, Katsaros D, Gimotty PA, Massobrio M, Regnani G, Makrigiannakis A, Gray H, Schlienger K, Liebman MN, Rubin SC, Coukos G (2003). Intratumoral T cells, recurrence, and survival in epithelial ovarian cancer. N Engl J Med.

[24] Patch AM, Christie EL, Etemadmoghadam D, Garsed DW, George J, Fereday S, Nones K, Cowin P, Alsop K, Bailey PJ, Kassahn KS, Newell F, Quinn MC (2015). Whole-genome characterization of chemoresistant ovarian cancer. Nature.

[25] Herbst RS, Soria JC, Kowanetz M, Fine GD, Hamid O, Gordon MS, Sosman JA, McDermott DF, Powderly JD, Gettinger SN, Kohrt HE, Horn L, Lawrence DP (2014). Predictive correlates of response to the anti-PD-L1 antibody MPDL3280A in cancer patients. Nature.

[26] Soslow RA, Han G, Park KJ, Garg K, Olvera N, Spriggs DR, Kauff ND, Levine DA (2011). Morphologic patterns associated with BRCA1 and BRCA2 genotype in ovarian carcinoma. Mod Pathol.

[27] Clarke B, Tinker AV, Lee CH, Subramanian S, van de Rijn M, Turbin D, Kalloger S, Han G, Ceballos K, Cadungog MG, Huntsman DG, Coukos G, Gilks CB (2009). Intraepithelial T cells and prognosis in ovarian carcinoma: novel associations with stage, tumor type, and BRCA1 loss. Mod Pathol.

[28] George J, Alsop K, Etemadmoghadam D, Hondow H, Mikeska T, Dobrovic A, deFazio A, Smyth GK, Levine DA, Mitchell G, Bowtell DD (2013). Nonequivalent gene expression and copy number alterations in high-grade serous ovarian cancers with BRCA1 and BRCA2 mutations. Clin Cancer Res.

[29] Sato E, Olson SH, Ahn J, Bundy B, Nishikawa H, Qian F, Jungbluth AA, Frosina D, Gnjatic S, Ambrosone C, Kepner J, Odunsi T, Ritter G (2005). Intraepithelial CD8+ tumor-infiltrating lymphocytes and a high CD8+/regulatory T cell ratio are associated with favorable prognosis in ovarian cancer. Proc Natl Acad Sci U S A.

[30] Rajasagi M, Shukla SA, Fritsch EF, Keskin DB, DeLuca D, Carmona E, Zhang W, Sougnez C, Cibulskis K, Sidney J, Stevenson K, Ritz J, Neuberg D (2014). Systematic identification of personal tumor-specific neoantigens in chronic lymphocytic leukemia. Blood.

[31] Shukla SA, Rooney MS, Rajasagi M, Tiao G, Dixon PM, Lawrence MS, Stevens J, Lane WJ, Dellagatta JL, Steelman S, Sougnez C, Cibulskis K, Kiezun A (2015). Comprehensive analysis of cancer-associated somatic mutations in class I HLA genes. Nat Biotechnol.

[32] Lawrence MS, Stojanov P, Mermel CH, Robinson JT, Garraway LA, Golub TR, Meyerson M, Gabriel SB, Lander ES, Getz G (2014). Discovery and saturation analysis of cancer genes across 21 tumour types. Nature.

[33] Wagle N, Berger MF, Davis MJ, Blumenstiel B, Defelice M, Pochanard P, Ducar M, Van Hummelen P, Macconaill LE, Hahn WC, Meyerson M, Gabriel SB, Garraway LA (2012). High-throughput detection of actionable genomic alterations in clinical tumor samples by targeted, massively parallel sequencing. Cancer Discov.

[34] Hamanishi J, Mandai M, Iwasaki M, Okazaki T, Tanaka Y, Yamaguchi K, Higuchi T, Yagi H, Takakura K, Minato N, Honjo T, Fujii S (2007). Programmed cell death 1 ligand 1 and tumor-infiltrating CD8+ T lymphocytes are prognostic factors of human ovarian cancer. Proc Natl Acad Sci U S A.

