# Supplementary material for: Association and prognostic significance of BRCA1/2-mutation status with neoantigen load, number of tumor-infiltrating lymphocytes and expression of PD-1/PD-L1 in high grade serous ovarian cancer
Source: Oncotarget. 2016 Feb 9;7(12):13587–98. doi: 10.18632/oncotarget.7277 (PMC4924663; doi:10.18632/oncotarget.7277)
Supplement: Supplementary file 1 [file oncotarget-07-13587-s001.pdf]

Association and prognostic significance of BRCA1/2-mutation status with neoantigen load, number of tumor-infiltrating lymphocytes and expression of PD-1/PD-L1 in high grade serous ovarian cancer

Supplementary Materials

Supplementary Table S1: Multivariate analyses including BRCA1/2-mutation status and neoantigen load in the TCGA dataset

Factor	HR	Cox *p*
BRCA1/2-mutated vs rest	0.479, 95% C.I. = 0.308–0.745	0.001
Neoantigen load (low quartile vs rest, cut-off 27)		0.232 (NS)

Factor	HR	Cox *p*
BRCA1/2-mutated vs rest	0.478, 95% C.I. = 0.308–0.741	0.001
Neoantigen load (low quintile vs rest, cut-off 25)		0.13 (NS)

Supplementary Table S2: Antibodies used in the study

Antibody	Company	Clone	Dilution	Antigen Retrieval Buffer
CD3	Dako	A0452 (polyclonal)	1:250	H2
CD4	Dako	M7310 (4B12)	1:80	H2
CD8	Dako	M7103 (C8/144B)	1:100	H2
CD20	Dako	M0755 (L26)	1:500	H1
PD-1	Cell Marque	315 m-95 (NAT105)	1:300	H2
PD-L1	Cell Signaling	13684 (E1L3N)	1:200	H2

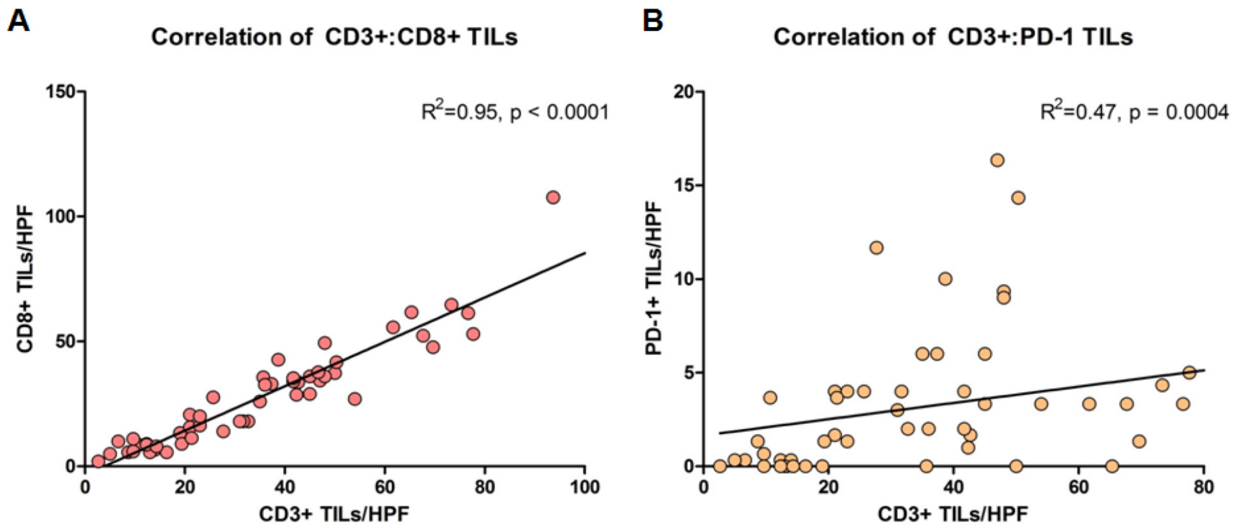

Supplementary Figure S1: Spearman correlation of CD3 and CD8 (A) and CD3 and PD-1 (B).

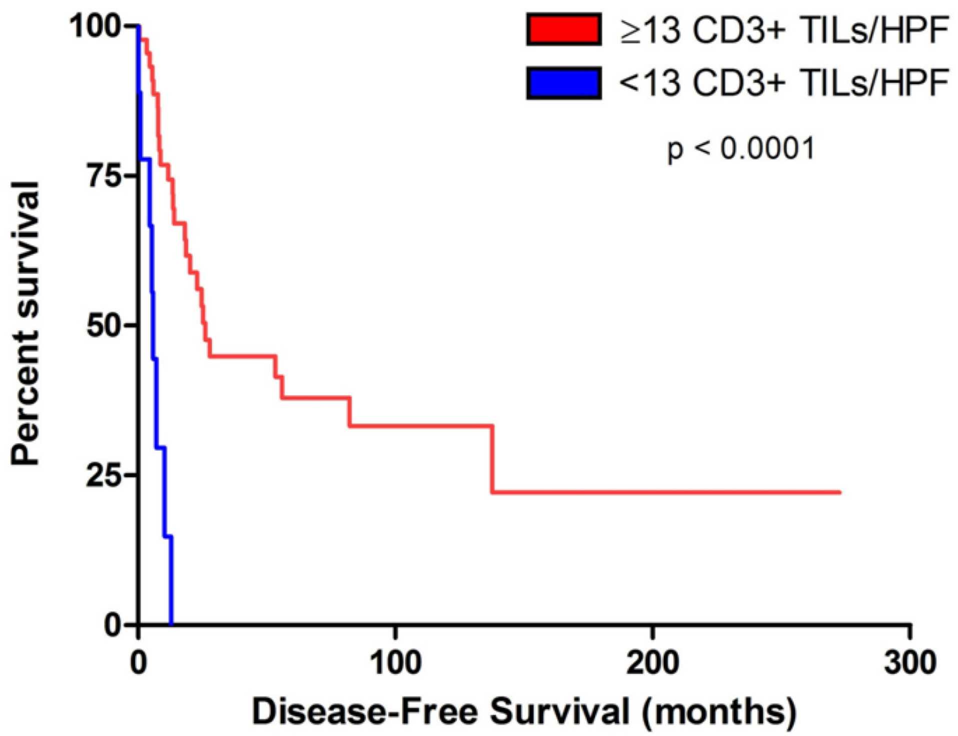

Supplementary Figure S2: Disease free survival for tumors with ≥ 13 CD3+ TILs/HPF versus tumors with < 13 CD3+ TILs per HPF.
